# THE HIGHWAY OF LIFE: SOCIAL VIRTUAL REALITY AS A REMINISCENCE TOOL

**DOI:** 10.1093/geroni/igz038.1121

**Published:** 2019-11-08

**Authors:** Steven J Baker, Jenny Waycott, Jeni Warburton, Frances Batchelor

**Affiliations:** 1 The University of Melbourne, Melbourne, Victoria, Australia; 2 La Trobe University, Wodonga, Victoria, Australia

## Abstract

A large body of research demonstrates the positive impact that reminiscence activities can have on older adult wellbeing. Within this space, researchers have begun to explore how virtual reality (VR) technology might be used as a reminiscence tool. The immersive characteristics of VR could aid reminiscence by giving the sense of being fully present in a virtual environment that evokes the time being explored in the reminiscence session. However, to date, research into the use of VR as a reminiscence tool has overwhelmingly focussed on static environments that can only be viewed by a single user. This paper reports on a first-of-its-kind research project that used social VR (multiple users co-present in a single virtual environment), and 3D representations of personal artifacts (such as, photographs and recorded anecdotes), to allow a group of older adults to reminisce about their school experiences. Sixteen older adults aged 70-81 participated in a four-month user study, meeting in groups with a facilitator in a social virtual world called the Highway of Life. Results demonstrate how the social experience, tailored environment, and personal artifacts that were features of the social VR environment allowed the older adults to collaboratively reminisce about their school days. We conclude by considering the benefits and challenges associated with using social VR as a reminiscence tool with older adults.

